# Prevalence of dengue and chikungunya virus infections in north-eastern Tanzania: a cross sectional study among participants presenting with malaria-like symptoms

**DOI:** 10.1186/s12879-016-1511-5

**Published:** 2016-04-26

**Authors:** Debora C. Kajeguka, Robert D. Kaaya, Steven Mwakalinga, Rogathe Ndossi, Arnold Ndaro, Jaffu O. Chilongola, Franklin W. Mosha, Karin L. Schiøler, Reginald A. Kavishe, Michael Alifrangis

**Affiliations:** Faculty of Medicine, Kilimanjaro Christian Medical University College, Moshi, Kilimanjaro, Tanzania; Centre for Medical Parasitology, Department Immunology and Microbiology, University of Copenhagen, Copenhagen, Denmark; Kilimanjaro Clinical research institute, Moshi, Kilimanjaro, Tanzania; Global Health Section, Department of Public Health, University of Copenhagen, Copenhagen, Denmark

**Keywords:** Dengue, Chikungunya, Arbovirus, Prevalence, Tanzania

## Abstract

**Background:**

In spite of increasing reports of dengue and chikungunya activity in Tanzania, limited research has been done to document the general epidemiology of dengue and chikungunya in the country. This study aimed at determining the sero-prevalence and prevalence of acute infections of dengue and chikungunya virus among participants presenting with malaria-like symptoms (fever, headache, rash, vomit, and joint pain) in three communities with distinct ecologies of north-eastern Tanzania.

**Methods:**

Cross sectional studies were conducted among 1100 participants (aged 2–70 years) presenting with malaria-like symptoms at health facilities at Bondo dispensary (Bondo, Tanga), Hai hospital (Hai, Kilimanjaro) and TPC hospital (Lower Moshi). Participants who were malaria negative using rapid diagnostic tests (mRDT) were screened for sero-positivity towards dengue and chikungunya Immunoglobulin G and M (IgG and IgM) using ELISA-based kits. Participants with specific symptoms defined as probable dengue and/or chikungunya by WHO (fever and various combinations of symptoms such as headache, rash, nausea/vomit, and joint pain) were further screened for acute dengue and chikungunya infections by PCR.

**Results:**

Out of a total of 1100 participants recruited, 91.2 % (*n =* 1003) were malaria negative by mRDT. Out of these, few of the participants (<5 %) were dengue IgM or IgG positive. A total of 381 participants had fever out of which 8.7 % (33/381) met the defined criteria for probable dengue, though none (0 %) was confirmed to be acute cases. Chikungunya IgM positives among febrile participants were 12.9 % (49/381) while IgG positives were at 3.7 % (14/381). A total of 74.2 % (283/381) participants met the defined criteria for probable chikungunya and 4.2 % (11/263) were confirmed by PCR to be acute chikungunya cases. Further analyses revealed that headache and joint pain were significantly associated with chikungunya IgM seropositivity.

**Conclusion:**

In north-eastern Tanzania, mainly chikungunya virus appears to be actively circulating in the population. Continuous surveillance is needed to determine the contribution of viral infections of fever cases. A possible establishment of arboviral vector preventive control measures and better diagnosis of pathogens to avoid over-treatment of other diseases should be considered.

**Electronic supplementary material:**

The online version of this article (doi:10.1186/s12879-016-1511-5) contains supplementary material, which is available to authorized users.

## Background

Dengue and chikungunya are major global health concerns due to their continued spread and intensifying epidemic activities throughout most of the tropical and subtropical regions of the world. Regular outbreaks of dengue have been reported throughout most of the tropical and sub-tropical regions of the world for several decades [1–3] while chikungunya epidemics have attained global distribution within the past ten years; yet large-scale epidemics of dengue fever and chikungunya fever has only recently presented as an emerging phenomenon in Africa [4].

These viruses are transmitted between humans by the bite of infected *Aedes aegypti* mosquitoes, furthermore chikungunya has increased its geographical range by its ability to infect *Aedes albopictus* [5, 6]. Most arbovirus infections are asymptomatic [7], but can as well cause a broad spectrum of manifestations, ranging from nonspecific flu-like syndrome to severe complications [8, 9].

Tanzania, as many other African countries, is experiencing a shift in disease transmission patterns with regards to febrile infectious diseases. While the number of cases of malaria are decreasing in several countries of sub-Saharan Africa [10–13], the number of febrile cases due to other causes than malaria are still high [14]. Various arboviruses are most likely important and are gaining more prominence as observed through an increase in reported activity [15–18] and epidemic activity of diseases such as dengue and chikungunya [1, 19–21]. Moreover, malaria may as well have been over-diagnosed in different parts of Tanzania [22–24], mainly due to lack of differential pathogen diagnosis apart from malaria [25], including dengue and chikungunya. Recent studies in Tanzania have revealed that patients with acute dengue and chikungunya infection are often misdiagnosed and treated with anti-malarials or antibiotics [22, 26]. The consequences of misdiagnosis and underreporting of other diseases than malaria include economic loss [27], development of drug resistant malaria strains (due to over-prescribing of antimalarials) [28] and risks of increased morbidity and mortality [29].

Little is known about the general epidemiology of dengue and chikungunya in Africa and Tanzania in particular. Thus, the objective of this study was to determine the sero-prevalence and as well, prevalence of acute infections of dengue and chikungunya virus among participants presenting with malaria-like symptoms (fever, headache, rash, vomit, and joint pain) in three communities with distinct ecologies of North-eastern Tanzania.

## Methods

### Study sites

We conducted cross sectional studies at three sites in north-eastern Tanzania; Bondo dispensary (Bondo, Tanga), Hai hospital (Hai, Kilimanjaro) and TPC hospital (Lower Moshi) in May 2013, November 2013 and May 2014, respectively. These sites were selected based on variation in precipitation pattern and varying altitude between the sites resulting in difference in malaria transmission, and as well, evidence of overdiagnosis of malaria [22]. Since most malaria and arbovirus transmission occur during the rainy seasons as a results of increased vector abundance, we chose to conduct the surveys during the rainy seasons that is in May and November. That is also the time when over diagnosis of malaria is most likely to occur. Bondo is a coastal lowland rural area in Handeni, Tanga region located 309 m above sea level. Bondo is endemic to malaria with a perennial transmission [30]. Hai is a sub-urban area situated on the South-western slope of Mount Kilimanjaro surrounded by three altitude zones ranging from highland (1,666–1,800 m), middle **(**900–1666 m) and lowland zone **(**900 m) above sea level. In Hai malaria transmission is classified as moderately high in lower altitude, moderate in mid-altitude and non-endemic in highland areas [31]. Lower Moshi is a lowland rural area located at the base of Mount Kilimanjaro at an altitude of 800 m above sea level. In Lower Moshi, malaria transmission occurs throughout the year [32, 33].

### Participant’s recruitment and data collection

Community sensitization to participate in the study was done through community leaders. Inhabitants aged 2 to 70 years with specific symptoms (fever, headache, rash, vomit, and joint pain) were encouraged to voluntarily go for testing at the health facilities at Bondo Dispensary Hai Hospital or TPC hospital. Individuals presenting at the health facilities were informed of the study and on voluntary participation. A written informed consent was obtained from all participants and from parents or guardians for children under 18 years of age who agreed to participate in the study. A questionnaire was used to collect demographic and clinical data such as age, gender and history of illness for each of the participants. All participants were screened for malaria by rapid diagnostic tests (SD BIOLINE Malaria Ag P.f/Pan) and malaria positive patients were treated with antimalarial according to national guidelines. In addition, auxiliary temperature measurement was done to detect febrile cases (>37.5 0C) and a blood sample of 0.5-1 mL was taken from all consenting participants. A subject number was assigned to each participant and used to label blood samples. All samples collected in this study were not linked to personal identifier.

### IgG and IgM ELISA for detection of anti-dengue and anti-chikungunya antibodies

Serum was separated from whole blood by centrifugation and stored at −20 °C. Anti-dengue IgM and IgG were detected using a direct enzyme linked immunosorbent assay (ELISA) kit (SD Inc, Gyeonggi-do, Korea) while anti-chikungunya IgM and IgG were analysed using Indirect Elisa kits (SD, Gyeonggi-do, Korea and IBL international, Hamburg, Germany, respectively). All assays were performed according to the manufacturers’ procedures and all serum samples were diluted 1 into 100 with sample diluent provided with the kits. The optical density (OD) was measured at 450 nm and the units of antibody concentration and cut-off values calculated as described by the manufacturers. Briefly, for the Anti-dengue IgM/IgG and IgM anti-chikungunya ELISAs the diagnostic cut-off value was calculated as the average OD of negative controls + 0.300. For the IgG chikungunya ELISA the threshold for positivity was based on the OD cut-off value of the cut-of control + 10 %.

### RNA extraction, cDNA synthesis and PCR

Only serum samples from participants with specific symptoms (fever, headache, rash, nausea, and joint pain) who met World Health Organization (WHO) criteria [9, 34] for probable dengue and chikungunya definitions (see below) were further tested by polymerase chain reaction (PCR). Ribonucleic acid (RNA) was extracted from serum samples using the Boom method [35]. Briefly, 30 μl of silica was added and immediately vortexed for 5 s followed by shaking for 10 min and centrifuged at 12,000 g for 15 s. Supernatant was removed and silica pellets was washed twice with 1 ml of L2 buffer (120 g of guanidine thiocyanate {GuSCN} in 100 ml of 0.1 M Tris–HCl, pH 6.4), twice with 1 ml of 70 % ethanol and once with 1 ml of acetone. Silica pellets was dried at 56 °C for 10 min. RNA was eluted from the silica pellets in 50 μl of diethyl pyrocarbonate (DEPEC) treated water and incubated for 10 min at 56 °C. Sample was vortexed and centrifuged for 2 min at 12,000 g. 35 μl containing purified RNA was transferred to an eppendorf tube and stored at −20 °C prior to use. cDNA was synthesized using Superscript® VILO™ cDNA synthesis kit (Invitrogen, life technologies, USA) according to manufacturer’s instructions in a total volume of 20 μL containing 2 μL of 10X Superscript® Enzyme Mix, 4 μL of 5X VILO™ reaction Mix, 11 μL of DEPEC treated water (Ambion, USA) and 3 μL of extracted RNA. The reverse transcription programme involved incubation at 25 °C for 10 min, extension at 42 °C for 60 min and inactivation at 85 °C for 5 min. The resulting cDNA was stored in −20 °C for further use for dengue and chikungunya PCR.

Dengue PCR was conducted as previously described [36] with respect to dengue only and with minor modifications using TaqMan Gene expression master mix kit (Applied Biosystens, USA). Briefly; the reaction mixture was composed of 5 μl of TagMan Gene Expression master mix (Applied Biosystens, USA), 0.3 μl of each 0.25 μM forward and reverse primer, 0.14 μL of 0.125 μM dengue probe, 3.37 μl nuclease free water (NorgenBiotek, Canada), and 1 μl of cDNA template in a final volume of 10 μl. The amplification reaction was performed in a Rotor-Gene Q PCR machine (Qiagen, Germany) with an initial denaturation at 95 °C for 2 min followed by 45 cycles of denaturation at 95 °C for 10 s and annealing and extension at 60 °C for minute. All primers and probes (Table 1) were purchased from BiolegioInc, Netherlands.

**Table 1:** Primers and Probe sequences used in detection of dengue and chikungunya

Sequence name	Primer sequence (5′–3′)	Region, position	Digital object identifier (DOI)
DENV-R	GHRGAGACAGCAGGATCTCTG	3′UTR, 10674–10694	10.1016/S1995–7645(12)60055–8
DENV-F	GACTAGYGGTTAGAGGAGACC	3′UTR, 10520–10541	
DENV-P	Cy5-AAGGACTAGMGGTTAGWGGAGACCC-BHQ2	3′UTR, 10610–10634	
CHIKV-F	CGTGGTGTACAAAGGTGACG	E1, 10524	
CHIKV-R	ACG CCG GGTAGTTGACTATG	E1, 11170	10.1002/jmv.23406

The primers (Table 1) used for conventional chikungunya PCR were adopted from Reddy et al., [37] to amplify a 646 bp gene product in the chikungunya envelope region of E1 (genome position: 10524–11170). The reaction was carried out in a Bio-Rad tetrad 2, (Biorad, USA) in a 20 μl volume containing 1 μL of cDNA, 4 μL of 5X reaction buffer, 1.8 μL of 25 mM MgCl_2_, 1.8 μL of 10 μM dNTP, 0.5 μL of 20 μM of each primers, 0.3 μL of Go-Tag Flex DNA polymerase, and 10.3 μL of nuclease free water (NorgenBiotek, Canada). The PCR programme consisted of an initial denaturation at 95 °C for 2 min and 40 cycles of 30 s denaturation at 95 °C, 30 s annealing at 55 °C, 1 min elongation at 72 °C and final extension at 72 °C for 5 min. The amplified gene products were identified by their molecular weights analyzed by electrophoresis on a 1.5 % agarose gel stained with ethidium bromide and visualized under UV light.

Dengue primers and probeswereadopted from Pongsir et al., [36] for detection of dengue by real time PCR while for chikungunya the primers wereadopted from Reddy et al., [37] for chikungunya conventional PCR.

### Case definitions

Dengue and chikungunya cases were classified according to WHO [9, 34]. In brief, probable dengue was defined as participants with fever and any two of the following criteria: nausea, vomiting, rash, pains, tourniquet test positive, leukopenia and/or a positive IgM ELISA test. Probable chikungunya: a patient meeting both the clinical (a characteristic triad of fever, rash and joint manifestations plus the following: nausea, vomiting, headache and/or an IgM positive test by ELISA) and epidemiological criteria (residing or having visited epidemic areas). Confirmed acute dengue or chikungunya was defined as a positive PCR result. Prior exposure was defined as presence of anti-dengue or anti-chikungunya IgG or IgM in serum.

### Statistical analyses

Data were compiled and analyzed using SPSS 20.0 software (SPSS Inc., Chicago, IL, USA). Chi-square (*χ*2) was used to compare categorical data while Fisher’s exact test was used in cases when expected counts were less than 5. Association of chikungunya seropositivity and symptoms was analyses by logistic regression. A *P*-value < 0.05 was considered statistically significant. Descriptive statistics are presented as medians and ranges for continuous variables after checking for normality and as proportions for categorical data.

## Results

### Baseline characteristics

Among patients presenting at Hai hospital (Hai district, Kilimanjaro), TPC hospital (Lower Moshi, Kilimanjaro) and Bondo Dispensary (Handeni, Tanga), 1100 participants were recruited (*n =* 240, *n =* 549 and *n =* 311, respectively), of which 61.3 % (674) were female and 61.6 % (678) were adults (≥15 years) (Table 2). The median age was 24 years (range; 2 to 70 years), with males having a lower median age than females (median, 15.5 and 26 years, respectively). Generally, the malaria prevalence by mRDT was 8.8 % (97/1100) while the remaining 91.2 % (1003/1100) were malaria negative. The three sites varied significantly in malaria prevalence; among children (2 to 14 years), the malaria prevalence were highest in participating children attending TPC hospital with 25.4 % (15/59) followed by 14.1 % (44/311) in Bondo dispensary and 0 % in Hai hospital. Among adults (≥15 years), the malaria prevalence were likewise highest in TPC hospital at 13.9 % (35/252) followed by Bondo dispensary at 1.3 % (3/238) and none in Hai hospital.

**Table 2:** Baseline characteristics of the studied population (*N =* 1100)

		Variable %(*n*)			
Sites		Bondo	Hai	TPC	Total
		*n =* 549	*n =* 240	*n =* 311	*N =* 1100
Sex	Male	37.3 (205)	32.9 (79)	45.7 (142)	38.7 (426)
	Female	62.7 (344)	67.1 (161)	54.3 (169)	61.3 (674)
Age	Adults	56.6 (311)	21.7 (52)	19.0 (59)	38.4 (422)
	Children	43.4 (238)	78.3 (188)	81.0 (252)	61.6 (678)
Total Malaria (mRDT)					
Positive		8.6 (47)	0 (0)	16.1 (50)	8.8 (97)
Negative		91.4 (502)	0 (240)	83.9 (261)	91.2 (1003)
Malaria RDT		*n =* 311	*n =* 52	*n =* 59	*n =* 422
*In children - Positive		14.1 (44)	0 (0)	25.4 (15)	14.0 (59)
Negative		85.9 (267)	100 (52)	74.6 (44)	86.0 (363)
Malaria RDT		*n =* 238	*n =* 188	*n =* 252	*n =* 678
**In adults - Positive		1.3 (3)	0 (0)	13.9 (35)	5.6 (38)
Negative		98.7 (235)	100 (188)	86.1 (217)	94.4 (640)
^a^Fever in children		*n =* 267	*n =* 52	*n =* 44	*n =* 363
Positive		22.8 (61)	78.8 (41)	52.3 (23)	34.4 (125)
Negative		77.2 (206)	21.2 (11)	47.7 (21)	65.6 (238)
^a^Fever in adults		*n =* 235	*n =* 188	*n =* 217	*n =* 640
Positive		40.4 (95)	24.5 (46)	53.0 (115)	40.0 (256)
Negative		59.6 (140)	75.5 (142)	47.0 (102)	60.0 (384)

*p = 0.01

**p < 0.01

^a^All malaria positive cases were excluded

After excluding all malaria positive cases the prevalence of fever in children were highest in Hai hospital at 78.8 % (41/52), followed by TPC hospital at 52.3 % (23/44) and Bondo dispensary at 22.8 % (61/267). Among adults, the prevalence of fever were 53.0 % (115/217) at TPC hospital, followed by Bondo dispensary at 40.4 % (95/235) and lastly, in Hai hospital at 24.5 % (46/188).

### Sero-prevalence of dengue and chikungunya among febrile and afebrile participants

Among all febrile participants, the total prevalence of dengue IgM positives was 1.3 % (5/381) all of which were from participants residing nearby TPC hospital (3.6 % (5/138) (Table 3). The prevalence of Dengue IgG positives among febrile participants were as well low, only 1.3 % (2/156) in Bondo dispensary, 1.1 % (1/87) in Hai hospital and 1.4 % (2/138) in TPC hospital. Among afebrile participants, the total prevalence of dengue IgM positives was only 0.8 % (5/622) out of which 0.6 % (2/346), 0.7 % (1/153) and 1.6 % (2/123) positives were observed in samples from Bondo dispensary, Hai hospital and TPC hospital, respectively. Conversely, dengue IgG positives were also low at the three sites.

**Table 3:** Seroprevalence of dengue and chikungunya among febrile and afebrile participants in north-eastern Tanzania (*N =* 1003)

	Febrile *n* (%)				Afebrile *n* (%)			
Sites	Bondo	Hai	TPC	Total	Bondo	Hai	TPC	Total
	*n =* 156	*n =* 87	*n =* 138	*n =* 381	*n =* 346	*n =* 153	*n =* 123	*n =* 622
Dengue IgM	^a^0 (0)	0 (0)	3.6 (5)	1.3 (5)	^a^0.6 (2)	0.7 (1)	1.6 (2)	0.8 (5)
Dengue IgG	^a^1.3 (2)	1.1 (1)	1.4 (2)	1.3 (5)	^a^1.2 (4)	0.7 (1)	0 (0)	0.8 (5)
Chikungunya IgM	14.7 (23)	13.8 (12)	10.1 (14)	12.9 (49)	12.1 (42)	20.3 (31)	10.6 (13)	13.8 (86)
Chikungunya IgG	^a^1.9 (3)	6.9 (6)	3.6 (5)	3.7 (14)	3.2 (11)	6.5 (10)	11.4 (14)	5.6 (35)

*IgM* Immunoglobulin M, *IgG* Immunoglobulin G

^a^Difference was calculated by Fishers exact test

Generally, we observed high IgM sero-prevalence among all participants while no difference between children 14.0 % (51/363) and adults 13.1 % (84/640), (*χ*^2^ = 0.17; *p =* 0.6) and IgG sero-prevalence was low with difference between children 3.0 % (11/363) and adults 5.9 (38/640), (*χ*^2^ = 4.21; *p =* 0.04). The total number of chikungunya IgM positives among febrile participants were 12.9 % (49/381), and there were no difference observed between children 12.0 % (14/49) and adults 13.3 % (34/49) (*χ*^2^ = 0.12; *p =* 0.7). No apparent differences between the sites were observed; 14.7 % (23/156) in Bondo dispensary, 13.8 % (12/87) in Hai hospital and 10.1 % (14/138) in TPC hospital (*χ*^2^ = 1.46; *p =* 0.4), (Table 3). The total number of IgG positives among febrile participants was lower; at 3.7 % (14/381) divided into 1.9 % (3/156) in Bondo dispensary, 6.9 % (6/87) in Hai hospital and 3.6 % (5/138) in TPC hospital (Table 3). Total number of IgG positives among febrile participants was not different between children 4.8 % (6/14) and adults 3.1 % (8/14) (*χ*^2^ = 0.66; *p =* 0.4).

Among afebrile participants, the total number of chikungunya IgM positives was higher, as compared to the febrile participants, at 13.8 % (86/622), though this was not significantly different (*χ*^2^ = 0.189; *p =* 0.6). Furthermore, the number of IgM positives was significantly different between the sites; 12.1 % (42/346) in Bondo dispensary, 20.3 % (31/153) in Hai hospital and 10.6 % (13/123) in TPC hospital (*χ*^2^ = 7.24; *p =* 0.02). Likewise; the observed total chikungunya IgG positives was lower than the IgM positives, at 5.6 % (35/622) though with a significant difference between the sites; 3.2 % (11/346) in Bondo dispensary, 6.5 % (10/153) in Hai hospital and 11.4 % (14/123) in TPC hospital (*χ*^2^ = 11.81; *p =* 0.03), (Table 3).

### Probable dengue and chikungunya

Among all febrile participants, 8.7 % (33/381) and 74.2 % (283/381) cases met the WHO criteria for probable dengue and chikungunya respectively. None of the cases were positive for dengue while 4.2 % (11/263) were confirmed acute chikungunya cases by PCR. Ten of the acute chikungunya cases were from Bondo and a single case came from Hai. Two of these cases were children (2 to 14 years).

### Association of chikungunya seropositivity and symptoms

In uni-variate analysis results, headache and joint pain were significant predictors of chikungunya IgM seropositivity with OR = 3.03 (2.06–4.4), *p <* 0.01 and OR = 1.80 (1.25–2.59), *p <* 0.01 respectively. However when we combined headache and joint pain we did not observe any association, OR = 0.84 (0.55–1.26); *p =* 0.4. Joint pain were marginally associated with chikungunya IgG seropositivity OR = 0.51 (0.26–0.9), *p =* 0.05, Tables 4 and 5. Results of laboratory test among participants are presented in Additional file 1: Table 6.

**Table 4:** Association of symptoms and chikungunya IgM seropositivity

				Univariate analysis		Multivariate analysis	
Symptoms	*n*	Positive % (*n*)	Negative % (*n*)	OR (95 % CI)	*p*-value	OR (95 % CI)	*p*-value
Fever							
Yes	381	36.3 (49)	38.2 (332)	0.92 (0.63–1.34)	0.6	-	-
No	622	63.7 (86)	61.8 (536)	Reference			
Rash							
Yes	9	0 (0)	1.0 (9)	-	-	-	-
No	994	100 (135)	99.0 (859)	Reference			
Nausea							
Yes	83	7.4 (10)	8.4 (73)	0.87 (0.43–1.73)	0.6	-	-
No	920	92.6 (125)	91.6 (795)	Reference			
Headache							
Yes	451	68.1 (92)	41.1 (359)	3.03 (2.06–4.46)	<0.001	2.90 (1.88–4.48)	<0.001
No	522	31.9 (43)	58.6 (509)	Reference		Reference	
Joint pain							
Yes	381	50.4 (68)	36.1 (313)	1.80 (1.25–2.59)	0.002	1.09 (0.72–1.65)	0.6
No	622	49.6 (67)	63.9 (555)	Reference		Reference	

*OR* Odds ratio, *CI* Confident interval

**Table 5:** Association of symptoms and chikungunyaIg Gseropositivity

				Univariate analysis		Multivariate analysis	
Symptoms	*n*	Positive % (*n*)	Negative % (*n*)	OR (95 % CI)	*p*-value	OR (95 % CI)	*p*-value
Fever							
Yes	381	28.6 (14)	38.5 (367)	0.64 (0.34–1.20)	0.1	0.76 (0.39–1.47)	0.4
No	622	71.4 (35)	61.5 (587)	Reference		Reference	
Rash							
Yes	9	0 (0)	0.9 (9)	-	-	-	-
No	994	100 (49)	99.1 (945)	Reference			
Nausea							
Yes	83	6.1 (3)	8.4 (80)	0.71 (0.21–2.34)	0.5	-	-
No	920	93.9 (46)	91.6 (874)	Reference			
Headache							
Yes	451	40.8 (20)	45.2 (431)	0.83 (0.46–1.50)	0.5	-	-
No	522	59.2 (29)	54.8 (523)	Reference			
Joint pain							
Yes	381	24.5 (12)	38.7 (369)	0.51 (0.26–0.9)	0.05	0.56 (0.28–1.11)	0.1
No	622	75.5 (37)	61.3 (585)	Reference		Reference	

*OR* Odds ratio, *CI* Confidence interval

## Discussion

Diagnosis of infectious diseases in resource poor settings is a challenge. In malaria endemic areas most febrile illnesses have been and are treated as malaria cases due to lack of diagnostic capacity to properly rule-out malaria as a cause of fever and as well, identify alternative fever-causing pathogens [14]. As for malaria, diagnosis of dengue and chikungunya based on clinical presentation is difficult. Furthermore, similar presentations of signs and symptoms in the initial stage of illness of dengue and chikungunya have been a challenge to distinguish the two diseases clinically [38, 39]. Although, the vast majority of clinical dengue and chikungunya cases are self-limiting, however, in particularly diagnosis of patients with dengue is important for patient management in case of severe symptoms if done in a timely manner.

This study was conducted in three sites in northern Tanzania, each community have different characteristics in terms of varying altitude and thus, malaria transmission that may had identified acute cases of dengue and chikungunya infections and variations in prevalence of serological markers of dengue and chikungunya between the sites. Accordingly, the three sites differed in malaria prevalence with TPC hospital having higher malaria prevalence as compared to Bondo dispensary and Hai hospital as based on mRDT diagnosis.

We observed few dengue IgM positive cases among febrile participants in TPC hospital in Moshi (3.6 %) and there were no confirmed acute dengue cases by PCR at any sites. Evidence shows that viral RNA can be detected in patients from 0–7 days following the onset of symptoms, which corresponds roughly to the duration of fever while IgM is detected in 3 to 5 or more days after the onset of illness/symptoms in the majority of infected individuals [40]. Therefore, there is a possibility that dengue IgM positive but PCR negative samples were collected towards the end of the acute phase beyond the viral RNA detection phase. Furthermore, the lack of PCR confirmed cases in the study sites are suggestive of the IgM positive cases having been imported rather than locally transmitted. During the study, four cases who live in Moshi were reported from the KCMC hospital in Moshi, a few kilometres from the TPC study site, after having visited Dar es Salaam during a large-scale dengue outbreak in April-May, 2014. These cases were PCR-confirmed by our study team (unpublished data). The sampling period for the TPC site coincides somewhat with the dengue outbreak in Dar es Salaam.

Only 1.3 % of the participants were IgG positive to dengue infection. This is lower than the previously reported (10.7 %) in Moshi by Hertz et al. [26]. This can be explained by the fact that the study by Hertz et al. was based at a referral hospital receiving cases from the whole north-eastern Tanzania catchment area, while our study was conducted in health facilities serving local communities in each site. Although cross-reactivity with other flavivirus cannot be completely ruled-out, the sensitivity and specificity of the IgG ELISA kits used in this study are reported to be 99.2 % and 96.2 %, respectively when compared to the hemagglutination-inhibition method and thus it is unlikely that the observed results are due to cross reactivity with other flavi-viruses.

Conversely, we identified a high sero-prevalence of chikungunya IgM (12.9 %) among febrile participants out of which 12.0 % were among febrile children. This is the first report on chikungunya IgM positivity in the study sites. The proportion of chikungunya IgM seropositivity was comparable between children and adults thus indicating similar exposure among the two age groups. The 12 % seropositivity observed in children ismore than twice as high as previously reported by Chipwaza et al. [41] in Kilosa, Morogoro (4.7 %). This study found 5.6 % of afebrile participants presenting with prior exposure to chikungunya infection as measured by IgG positivity. Since there is no routine diagnosis for this virus at the health facilities in the sites, it is likely that such infections go unnoticed or when causing fever end up being treated as other common infections such as malaria or bacterial infections. Unexpectedly, we observed that the number of chikungunya IgG positives were lower than the number of IgM positives. While the IgM positivity did not differ among children and adults, IgG positivity was significantly higher in adults (5.9 %) than in children (3.0 %). This is indicative of active circulation of chikungunya in the study sites as supported by PCR results, with adults having prior exposure which also points to a possible endemicity and cumulative increases in IgG seropositivity with age [42].

We identified an overall prevalence of 4.2 % of acute chikungunya infections among study participants; with Bondo dispensary having the highest number of cases (*n =* 10, 2.0 %). This is somewhat lower than the previous reported in Kilimanjaro region of 7.9 % [22, 26] however, taken together it seems that chikungunya, most likely as opposed to dengue, was endemic and actively circulating in North-eastern Tanzania at the time of the study.

However, it is not known whether the chikungunya virus was silent or been actively present in Tanzania because no research has been done for the past 60 years since its discovery [43] to document its distribution. Symptoms of chikungunya infection last for approximately 5 days, during which is the time the virus is present in the blood circulation and can be detected by PCR. After the viremic phase, sensitivity of diagnosis of acute infection is minimal [44] as the level of virus declines. We have observed few PCR positive in Hai and none in TPC (Lower Moshi) and this could be due to timing of blood sampling in acute infection.

The present study showed a significant association between clinical symptoms (joint pain and headache) and the probability of positive diagnosis of chikungunya IgM. These results are in line with other studies, that reported headache, nausea, vomiting, myalgia, and rash were associated with chikungunya infection [45, 46]. We also observed association of chikungunya IgG seropositivity and joint pain. It is known that joint pain is one of the clinical features for chikungunya infection and its effect can persist for long [47–50]. However, these symptoms are common and non-specific, and thus could be mistakenly identified as caused by a variety of other febrile illnesses such as malaria, typhoid, dysentery, and bacterial meningitis [51, 52] and pose a challenge to differential diagnosis.

We did observe quite a low number of mRDT positives in the 3 sites; preliminary data suggest that, as expected, a fraction of the mRDT negatives are plasmodium positive when using a sensitive PCR method (data not shown), and therefore the low parasitaemi as are having no impact on fever/symptoms observed in participants, nevertheless after excluding all malaria positive, we found that 38.0 % of the remaining participants reported fever. It is known that in many malaria endemic areas where clear diagnosis cannot be established, all fevers may still be treated as malaria [53]. Therefore, we recommend establishment of better diagnosis of fever to avoid over-treatment of other diseases.

## Conclusions

This is the first seroprevalence report of dengue and chikungunya among participants presenting with malaria-like symptoms in three communities with distinct ecologies of in north-eastern Tanzania. Chikungunya virus appears to be actively circulating in the population, while dengue circulates in low level. Among malaria-like symptoms, headache was associated with chikungunya IgM seropositivity.

### Ethics approval and consent to participate

Ethical approval for this study was obtained from the Kilimanjaro Christian Medical University College Research and Ethics Review Committee (CRERC) with a certificate number 658. A written informed consent was obtained from all participants and from parents or guardians for children under 18 years of age who agreed to participate in the study.

## Additional file

Additional file 1: Table S6. Labororatory results among probable and non-probable dengue and chikungunya. (XLSX 112 kb)

## Abbreviations

BSU: building stronger university
CDC: centers for disease and control prevention
cDNA: complementary deoxyribo nucleic acid
CHIKV-F: chikungunya virus-forward
CHIKV-R: chikungunya virus-reverse
CRERC: College Research and Ethics Review Committee
DANIDA: Danish International Development Agency
DENV-F: dengue virus- forward
DENV-R: dengue virus- reverse
DEPEC: diethyl pyrocarbonate
DFC: Danida Fellowship Centre
ELISA: enzyme-linked immunosorbent assay
GuSCN: guanidine thiocyanate
IgG: immunoglobulin G
IgM: immunoglobulin M
MRDT: malaria rapid diagnostic test
OD: optical density
PCR: polymerase chain reaction
RNA: ribonucleic acid
SD: standard diagnostic
TPC: Tanzania plantations company
WHO: World Health Organization
